# Proteomic and Bioinformatics Analyses of Mouse Liver Microsomes

**DOI:** 10.1155/2012/832569

**Published:** 2012-03-20

**Authors:** Fang Peng, Xianquan Zhan, Mao-Yu Li, Fan Fang, Guoqing Li, Cui Li, Peng-Fei Zhang, Zhuchu Chen

**Affiliations:** ^1^Key Laboratory of Cancer Proteomics of Chinese Ministry of Health, Xiangya Hospital, Central South University, Hunan, Changsha 410008, China; ^2^Department of Biology, School of Pharmacy and Life Science, University of South China, Hengyang 421001, China

## Abstract

Microsomes are derived mostly from endoplasmic reticulum and are an ideal target to investigate compound metabolism, membrane-bound enzyme functions, lipid-protein interactions, and drug-drug interactions. To better understand the molecular mechanisms of the liver and its diseases, mouse liver microsomes were isolated and enriched with differential centrifugation and sucrose gradient centrifugation, and microsome membrane proteins were further extracted from isolated microsomal fractions by the carbonate method. The enriched microsome proteins were arrayed with two-dimensional gel electrophoresis (2DE) and carbonate-extracted microsome membrane proteins with one-dimensional gel electrophoresis (1DE). A total of 183 2DE-arrayed proteins and 99 1DE-separated proteins were identified with tandem mass spectrometry. A total of 259 nonredundant microsomal proteins were obtained and represent the proteomic profile of mouse liver microsomes, including 62 definite microsome membrane proteins. The comprehensive bioinformatics analyses revealed the functional categories of those microsome proteins and provided clues into biological functions of the liver. The systematic analyses of the proteomic profile of mouse liver microsomes not only reveal essential, valuable information about the biological function of the liver, but they also provide important reference data to analyze liver disease-related microsome proteins for biomarker discovery and mechanism clarification of liver disease.

## 1. Introduction

The liver, a vital organ, has a wide range of physiological functions and plays a major role in metabolism, biosynthesis, and chemical neutralizing. Liver diseases, such as viral hepatitis and liver cancer, pose a worldwide public health challenge. The Human Liver Proteome Project (HLPP) was launched in 2002 to better understand molecular liver functions and diseases, and liver proteome expression profile is one of the major parts of HLPP [1]. Because of the complexity, no single proteomic analysis strategy can sufficiently address all components of a proteome. Analysis of the subcellular proteome would provide insight into the functions of a given tissue or cell line. Subcellular proteomics reduces the complexity of a proteome [2, 3], detects some low-abundance proteins, and offers more detailed information that would contribute to the understanding of the function of the entire proteome.

Microsomes are composed primarily of closed sacs of membrane called vesicles that are derived mostly from endoplasmic reticulum (ER). As for liver, in addition to components of the protein secretary pathway, microsomes contain a multitude of proteins that are involved in lipid/lipoprotein biosynthesis and drug metabolism. The liver microsome is an ideal way to study the metabolism of compounds, the functional properties of membrane-bound enzymes, lipid-protein interactions, and drug-drug interactions [4, 5]. The proteomic profiling of the microsomes combined with bioinformatics analysis can reveal more essential information about the biological function of the liver. The main goal of this study was to systematically identify the protein components of the liver microsomes, to conduct the functional annotation with bioinformatics analysis, and to provide insight into the biological functions of the liver.

Two-dimensional gel electrophoresis (2DE) is one of the most widespread techniques for the proteomic profiling of soluble proteins and visualizes isoforms and posttranslational modifications in a proteome [6, 7]. Membrane proteins, however, are less amenable to solubilization in protein extraction buffers and are also susceptible to precipitation during isoelectric focusing (IEF) because of their hydrophobicity and alkaline pH value. One study showed that the analytical performance of one-dimensional gel electrophoresis (1DE) that separates endoplasmic reticulum membrane proteins is incomparably greater than that of 2DE [8]. Other studies [7, 9] demonstrated that the proteomic analysis of subcellular organelles, such as microsomes that contain a considerable number of highly hydrophobic membrane proteins, should be performed by combining 1DE and 2DE.

Although many of microsome proteins have been studied, many more remain to be isolated and characterized. With the improvement of current methodologies and experimental techniques, more proteomic data will be obtained. Also, biological interpretation of proteomic data and extracting biological knowledge are essential to further understanding liver function.

In our study, 2DE was first used to array the isolated microsome proteins of the liver. Because of the low performance of 2DE in separating membrane proteins [10] and the high efficiency of the carbonate procedure in separating membrane proteins [11, 12], the membrane proteins from Na_2_CO_3_-treated microsomes were separated by 1DE. Moreover, bioinformatics analysis of microsome proteomic data was performed to discover biological roles of the proteins. The results showed that the combination of 1DE and 2DE was more efficient for analyzing microsomes. Bioinformatics analysis can provide a valuable molecular basis to interpret the mechanisms underlying microsome biological functions and give insight into the biological function of the liver at the level of microsomes.

## 2. Material and Methods

### 2.1. Animals

 Male C57 mice (9 weeks old) were purchased from the Experimental Animal Center of Central South University (Changsha, China). The mice were starved overnight for liver subcellular fractionation. All experiments were performed with the approval of the institutional ethics committee on animal research.

### 2.2. Preparation, Validation, and 2DE Analysis of Microsomes

#### 2.2.1. Preparation of Microsomes

 Microsome apparatus-rich fractions were prepared from mice livers with differential centrifugation and sucrose gradient centrifugation as described [13]. Mice livers (approximately 10 g each) were drained of blood, minced thoroughly with scalpels, and transferred to 50 mL of chilled homogenization medium (0.25 M sucrose, pH 7.4) for 5–10 min with occasional stirring. The liquid was decanted and replaced with 50 mL of fresh homogenization medium followed by homogenization (30–60 sec.) on a TAMATO homogenizer (1,000 rpm × 3 and 1,500 rpm × 3). The homogenate was squeezed through a single layer of microcloth and centrifuged (10 min, 1,000 g; HITACHI centrifuge). The supernatant was centrifuged (30 min, 3,000 g), and sequentially centrifuged (30 min, 8,000 g) after discarding the sediment. The remainder supernatant was centrifuged (30 min, 34,000 g), carefully decanted, and centrifuged again (130,000 g, 1 h; Beckman Instruments, Palo Alto, CA) to get the “light” microsomes. The pink sediment was gently resuspended with a glass homogenizer in ~7 mL of 52% sucrose-0.1 M H_3_PO_4_ buffer (pH 7.1), and the density of sucrose was adjusted to 43.7%. The fraction was placed in one type-70i rotor centrifuge tube; overlayered sequentially with 7 mL, 5 mL, 5 mL, and 6 mL of 38.7%, 36.0%, 33.0%, and 29.0% sucrose, respectively, and centrifuged (80,000 g, 1 h). The upper four layers of the sucrose gradient were discarded by aspiration, and the bottom layer (43.7%) was diluted with two volumes of cold distilled water and centrifuged (130,000 g, 1 h) in a type-70i rotor to get the “heavy” microsomes. The pellets, light and heavy microsomes, were suspended in 3 mL of 0.25 M sucrose (pH 7.0) and combined. The mixture was diluted to 14 mL with 0.25 M sucrose containing CsCl with its final concentration of 0.015 M. The suspension was layered into an equal volume of 1.3 M sucrose/0.015 M CsCl and then centrifuged (240,000 g, 1 h) in an SW 55Ti rotor. The rough microsomes were in the pink sediment, and the smooth microsomes were at the interface. The smooth microsomes were diluted with an equal volume of 0.25 M sucrose (pH 7.0) and centrifuged (140,000 g, 1 h) in an SW 55i rotor.

#### 2.2.2. Detection and Validation of the Purity of Microsomes

Electron microscopy and Western blotting were used to detect and validate the purity of prepared microsomes. For electron microscope analysis, the prepared microsomes were fixed with 2.5% glutaraldehyde for 24 h and 2% OsO4 for 2 h, dehydrated with alcohol (50%, 70%, 90%, and 100% in turn), and processed into epoxy resin. Thin sections (500 Å*‌*) were prepared and stained with uranyl acetate and lead citrate then examined with a transmission electron microscope (H-600-1, Hitachi, Japan). For Western blotting analysis, the microsome fractions were lysed (4°C; 30 min) in lysis buffer (50 mM Tris-Hcl, 150 mM NaCl, 1 mM EDTA, 1% Triton-X100, and 0.1% SDS). The protein samples (50 *μ*g) were subjected to electrophoresis on SDS-PAGE with 12% gel and transferred to PVDF membrane (Millipore). The PVDF membranes with proteins were immunoblotted with antibodies to endoplasmin (ER marker), OxPhos complex IV subunit I (mitochondrial marker), catalase (peroxisomal marker), and cadherin (cytoplasmic marker), respectively.

#### 2.2.3. Separation of Microsome Proteins by 2DE

 2DE was performed as described by the manufacturer (Amersham Biosciences). Protein samples (400 *μ*g) were diluted to 450 *μ*L with rehydration solution (7 mol/L urea, 2 mol/L thiourea, 0.2% DTT, 0.5% (v/v) pH3–10 NL IPG buffer, and trace bromophenol blue) and applied to IPG strips (pH 3–10 NL; 24 cm) for rehydration (14 h; 30 V). Proteins were focused successively (1 h at 500 V, 1 h at 1,000 V, and 8.5 h at 8,000 V) to give a total of 68 kVh on an IPGphor. After equilibration, SDS-PAGE was performed with 12% gel on Ettan DALT II system. Then, the blue silver staining method was used to visualize the protein spots on the 2DE gels [14].

### 2.3. Na_2_CO_3_ Extraction and 1DE Analysis of Microsome Membrane Proteins

 Microsome membrane proteins were further extracted by the carbonate method from isolated microsomal fractions [12]. Microsomal fractions were diluted 50- to 1,000-fold with 100 mM sodium carbonate (pH 11.5; final protein concentration to 0.02 to 1 mg/mL), and incubated (0°C; 30 min) with slow stirring and accompanying sonication for 15 sec at 3-4 W at 0 min, 15 min, and 30 min. The suspensions were centrifuged and decanted, and the membrane pellets were gently rinsed three times with ice-cold distilled water. These pellets were diluted with denaturing sample buffer (5% mercaptoethanol, 2% SDS, 0.06 M Tris-HCl, pH 6.8, and 10% glycerol), heated (95°C; 5 min), and then subjected to 1D SDS-PAGE with a 12% gel. Electrophoresis was performed at 80 V for 20 min, followed by 100 V for 2 h. Gels were visualized with Coomassie Brilliant Blue G [14].

### 2.4. Tandem Mass Spectrometry (MS/MS) Identification of Proteins

#### 2.4.1. In-Gel Digestion

 The proteins contained in the 2D gel spots and 1D gel bands were subjected to in-gel digestion with trypsin. Gel spots or bands were excised and destained with 100 mM NH_4_HCO_3_ in 50% acetonitrile (ACN) at room temperature. The proteins were reduced with 10 mM dithiothreitol (DDT) (56°C; 30 min) and alkylated with 50 mM iodoacetamide in 100 mM NH_4_HCO_3_ (dark, room temperature, 30 min). The gel pieces that contained proteins were dried and then incubated in the digestion solution (40 mM NH_4_HCO_3_, 9% ACN, and 20 *μ*g/mL trypsin; 18 h, 37°C). The tryptic peptides were extracted with 50% ACN/2.5% TFA and then dried using a Speed-Vac.

#### 2.4.2. Nanoliquid Chromatography (LC) MS/MS and Protein Identification

The tryptic peptide mixture was fractionated with reverse-phase (RP) high-performance liquid chromatography (HPLC) by using an Ultimate nano-HPLC system (Dionex). Peptide samples were purified and concentrated with a C18-PepMap precolumn and then separated on an analytical C18-PepMap column (75 *μ*m ID × 150 mm, 100 Å pore size, 3 mm particle size) at a column flow rate of 300 nL/min. The ACN gradient (solution A: 0.1% formic acid, 2% ACN; solution B: 0.1% formic acid, 80% ACN) started at 5% B and ended at 70% B in 45 min. Mass spectrometry (MS) and MS/MS data were acquired using a Micromass quadrupole time of flight Micromass spectrometer (Waters). Database searches were carried out with the MASCOT server by using a decoy database (concatenated forward-reverse mouse IPI database, version 3.07; release date June 20, 2005). A mass tolerance of 0.3 Da for both parent (MS) and fragmented (MS/MS) ions, allowance for up to one trypsin miscleavage, variable amino acid modifications consisting of methionine oxidation and cysteine carbamidomethylation were used. MS/MS ion score threshold was determined to produce a false-positive rate less than 5% for a significant hit (**P** < 0.05). The false-positive rate was calculated with 2* reverse/(reverse + forward)/100. In the current study, the MS/MS ion score threshold was 23 and a false-positive rate was approximately 3.1%. For all the proteins that were identified with only one peptide, each MS/MS spectrum was checked manually.

### 2.5. Bioinformatics Analysis of Identified Proteins

 Protein annotations were obtained primarily from UniProt 7.0 including accession, entry name, comments such as function, catalytic activity, subcellular location, and similarity. The Cytoscape plugin, Biological Networks Gene Ontology (BinGO), was used to find statistically overrepresented GO categories of the protein dataset. An online tool, WebGestalt (http://bioinfo.vanderbilt.edu/webgestalt/), was used to map target proteins to Kyoto Encyclopedia of Genes and Genomes (KEGG) pathways. The pathway visualization was based on the pathway mapping service provided in KEGG.

## 3. Results

### 3.1. Characterization and Detection of Liver Microsomes

It was essential to obtain a highly pure fraction to conduct proteomic characterization of microsomes. The purity of prepared microsomes was monitored with electron microscope and Western blotting analysis. A large number of nearly spherical membrane vesicles were visualized with electron microscope without other contaminated organelle compositions (see Supplemental Figure  1(a) in Supplementary Material available online at doi:10.1155/2012/832569). Western blotting analyses showed that, with the standard immunoblotting protocol, the ER marker endoplasmin was enriched in the isolated microsome fractions without the contamination marker (mitochondrial marker OxPhos Complex IV subunit I, peroxisomal marker catalase, and cytoplasmic marker cadherin) being detected (Supplemental Figure  1(b)). The results demonstrated an optimized preparation of microsomes. 

### 3.2. Fractionation and Identification of Microsome Proteins Identified by 2DE and MS/MS

**Figure 1:**
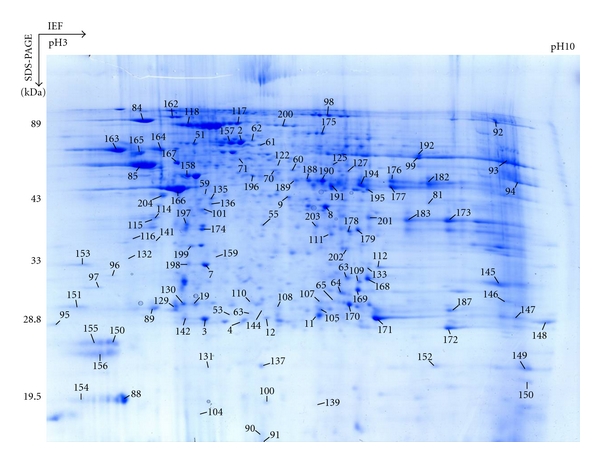
2DE pattern of mouse liver microsome. Microsomal proteins (400 *μ*g) were arrayed by 2DE with IPG strip (pH 3–10 NL; 24 cm) and SDS-PAGE with 12% gel and visualized with blue silver staining method. A total of 204 spots denoted by circles were MS-analyzed.

The 2DE reference maps display  514 ± 83  protein spots (*n* = 10 gels). A representative 2DE map of microsome proteins was shown (Figure 1). A total of 183 proteins were identified with ESI-Q-TOF MS/MS from 204 excised gel spots. Those proteins are summarized (Table 1 and Supplemental Table  1), including 2D gel-spot number, IPI number, protein name, predicted TMD, and subcellular location. The microsomal marker proteins such as endoplasmin (Spot 2) and UDP glucuronosyltransferase (Spots 6 and 7) were identified. Those proteins were located in different subcellular locations (Table 1) including ER, mitochondrial membrane, cytoplasmic, ribosome, microbody, microsome membrane, nuclear, vesicular membrane, sarcolemma, extracellular space, cilium, ER-Golgi intermediate compartment, and secreted proteins. Supplemental Figure  2 shows the percentage of each group of proteins, according to their subcellular locations, derived from the annotations in the Swiss-Prot database and Gene Ontology: 22% of proteins (*n* = 41) from ER and Golgi, 11% of proteins (*n* = 20) from mitochondria and other membranes, 50% of proteins (*n* = 91) from cytosolic and other soluble proteins, 8% of secreted proteins (*n* = 15), and 9% of proteins without unambiguous location (*n* = 16).

### 3.3. Fractionation and Identification of Microsomal Membrane Proteins Identified by 1DE and MS/MS

**Figure 2:**
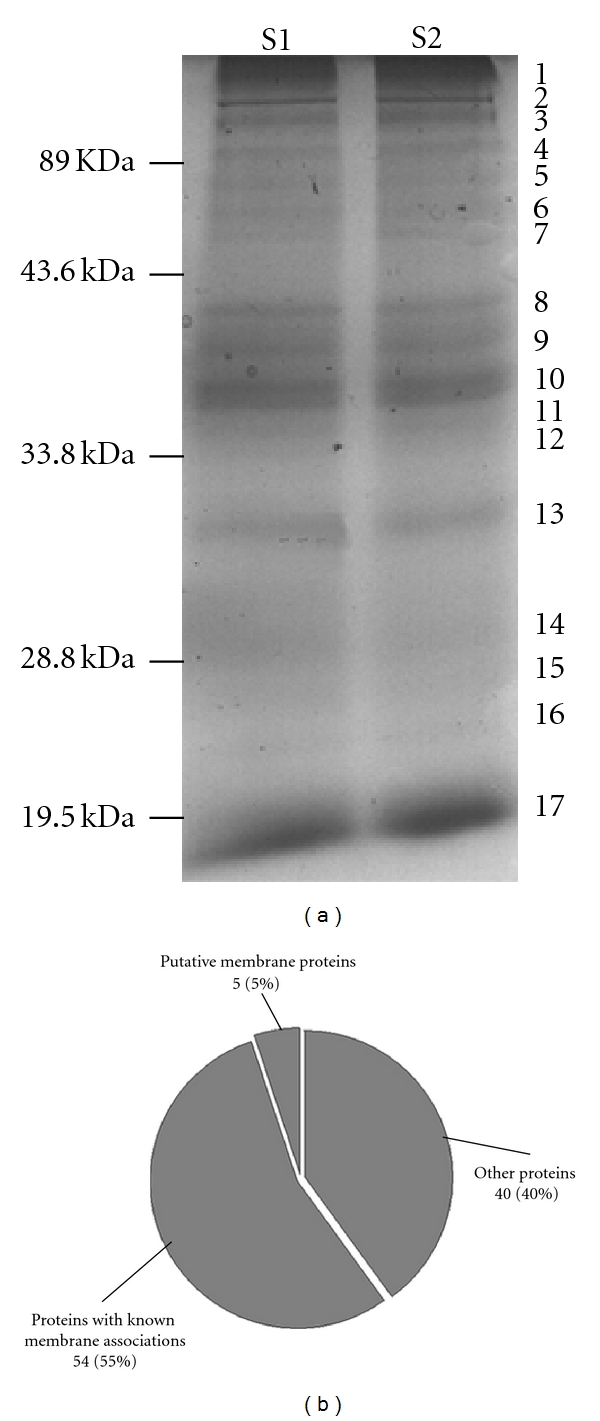
1DE pattern and membrane-associated characteristic classification of Na_2_CO_3_-extracted microsomal membrane proteins. (a) 1DE pattern. Molecular weight markers are shown on the left and bands excised for MS analysis are indicated on the right. Lanes S1 and S2 were loaded with the same protein samples (50 *μ*g per lane). (b) Classification via membrane-associated characteristic. The criteria used for this classification were published reports, annotations in the genome database (http://www.uniprot.org/), and predictions for transmembrane regions (http://www.cbs.dtu.dk/services/TMHMM/).

The Na_2_CO_3_-treated microsome membrane proteins were separated on SDS-PAGE gels and visualized with Coomassie brilliant blue staining (Figure 2(a)). A total of 99 proteins (Table 2 and Supplemental Table  2) was identified with electrospray ionization- (ESI-) Q-TOF MS/MS from 17 gel bands (Figure 2(a)). Those proteins were derived from the ER, type I/II membrane proteins, integral membrane proteins, major histocompatibility complex class I protein, ER-Golgi intermediate compartment, mitochondrial membrane, nuclear, cytoplasm, microbody, sarcolemma, and secreted and unknown proteins (Table 2). Those membrane proteins were classified into three categories (Figure 2(b)): (a) proteins with known membrane associations (55%; *n* = 54), (b) putative membrane proteins (5%; *n* = 5), and (c) other proteins (40%; *n* = 40). Those identified proteins were categorized according to the reported annotation in the UniProt database (http://www.uniprot.org/) and predictions for transmembrane regions (http://www.cbs.dtu.dk/services/TMHMM/). Of the 99 proteins, 59 (60%) were described as “membrane-associated” proteins (category (a) and (b)), including ER-characteristic proteins (cytochromes P-450 and b5, calnexin, integral membrane enzymes such as NADPH-cytochrome c reductase, and microsomal glutathione S-transferase 1).

Hydrophobicity is an important characteristic of a membrane protein. The grand average of hydropathy (GRAVY) scores (>−0.4) (http://us.expasy.org/tools/protparam.html) is an index to evaluate the hydrophobic status of a protein, indicates a hydrophobic protein, and suggests a membrane association. In the current study, 69 (70%) of the 99 proteins identified from 1DE had a GRAVY > −0.4 (Supplemental Figure  3), a score indicating the probability for membrane association. Moreover, some alkaline proteins with *PI* values close to or greater than 10 were separated by 1DE (Supplemental Figure  4), but they could not be detected in a conventional 2DE map.

### 3.4. Comparison of 2DE and 1DE Datasets

Among the 2DE dataset (*n* = 183 proteins; Table 1) and 1DE dataset (*n* = 99 proteins; Table 2), only 23 proteins (Table 3) were consistent between 2DE and 1DE datasets (23% of 1DE dataset, and 13% of 2DE dataset). A total of 259 nonredundant proteins (*n* = 183 + 99 − 23) were identified in the microsome fraction through the strategy of combining 2DE with 1DE protein-separation technologies followed by ESI-Q-TOF MS/MS. The microsome consisted of a complex network of continuous membranes including ER, ER-Golgi intermediate complex—also referred to as the vesiculotubular clusters or pre-Golgi intermediates—and the Golgi apparatus [5]. Among those identified proteins, 62 located in ER and Golgi were definitely classified as microsome proteins by annotation in the Swiss-Prot database and the Gene Ontology (GO).

### 3.5. Significantly Enriched GO Terms for Mouse Liver Microsome Proteins

Biological Networks Gene Ontology [15] and Cytoscape [16] plugins to find statistically overrepresented GO categories were used for the enrichment analysis of our protein dataset. The microsome protein dataset (*n* = 259, from 1DE and 2DE datasets) was compared to a reference set of complete mouse proteome (IPI mouse) that was provided by Biological Networks Gene Ontology. The analysis was done with a hypergeometric test, and all significant (*P* < 0.01) GO terms were selected after correcting for a multiple term testing with a Benjamini and Hochberg false discovery rate. The analysis was performed separately for molecular function, cellular component, and biological process categories, and x-fold enrichment for every overrepresented term in three GO categories was calculated (Supplemental Figure  5). The results showed that the terms were related to mostly catalytic activity in terms of molecular function, including metabolism-related oxidoreductase, hydrolase, and dehydrogenase. Similarly, terms belonging to the cellular component namespace include mitochondrion, ER, and ribosome. Finally, terms from the biological process namespace included metabolic process, localization, transport, and translation. All of the information suggested the main functions and compositions of microsome.

### 3.6. Significant Enrichment of KEGG Pathway for Mouse Liver Microsome Proteins

**Figure 3:**
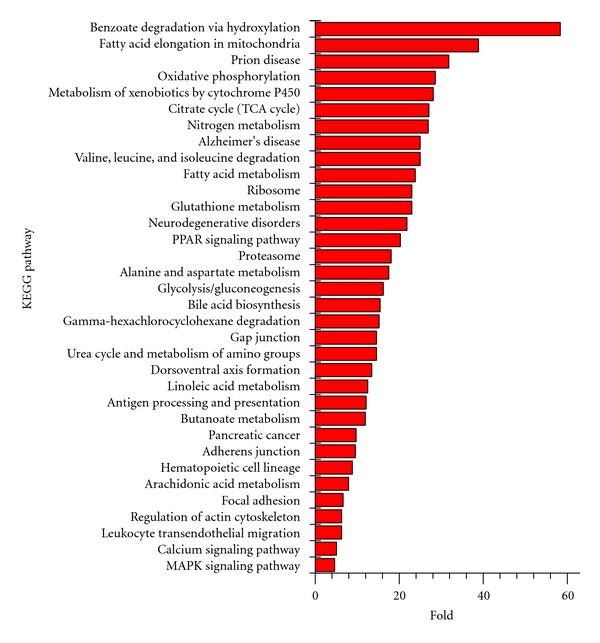
Significantly enriched KEGG pathways for mouse liver microsome proteins (*n* = 259) that were derived from 1DE and 2DE strategies. KEGG pathway enrichment analysis was performed using WebGestalt. The pathways having enrichment (*P* < 0.01) are presented. For each KEGG pathway, the bar shows the x-fold enrichment of the pathway in our dataset.

Biological pathways analysis based on KEGG pathway database was performed with an analysis toolkit—WebGestalt (http://bioinfo.vanderbilt.edu/webgestalt/) [17]. This toolkit allowed the functional annotation of gene/protein sets into well-characterized functional signaling pathways (KEGG: http://www.genome.jp/kegg/). In addition, an enrichment score was obtained of the frequency of occurrence of a specific protein (or gene) within any given experimental subset with respect to a species-specific background set. Thus, an enrichment factor (the observed frequency in input set/the expected frequency in background set) was created with a statistical value that indicated that the protein (or gene) was specifically overrepresented in the input dataset. In this current study, all the proteins except 81 (*n* = 259 − 81 = 178) were linked to a total of 99 biological pathways in the KEGG, including metabolic pathway, glycolysis/gluconeogenesis, metabolism of xenobiotics by cytochrome P450, and PPAR signaling pathway. Among those pathways, 34 significantly (*P* < 0.01) enriched biological processes analyzed by WebGestalt were obtained (Figure 3). Those biological processes were involved in cell metabolism, benzoate degradation, metabolism of xenobiotics, ribosome, biosynthesis, signaling pathway, and oxidative stress. Those results are known to be related to microsome.

**Figure 4:**
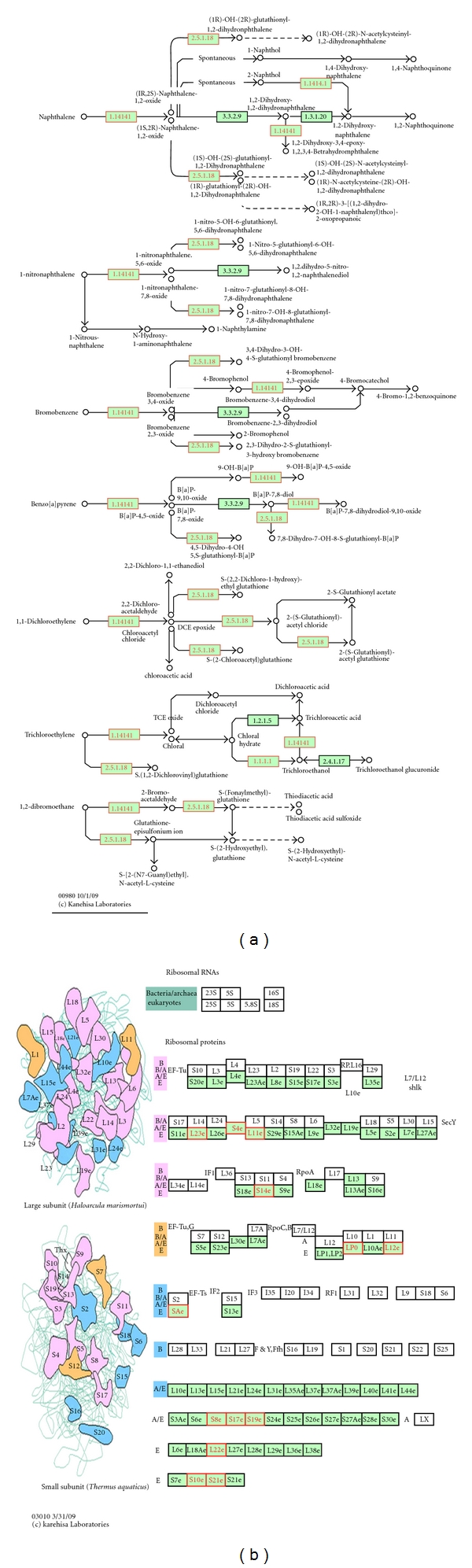
Metabolism of xenobiotics by cytochrome P450 pathway, and ribosome map views of identified proteins. The two enriched metabolic pathway maps were generated by KEGG, which incorporated the proteomic data into the KEGG pathway maps. All of the genes in mouse are colored; the genes contained in the protein dataset are red.

To ascertain the coverage of our dataset with the enriched pathways or biological processes, the KEGG search service was used to map our dataset on KEGG pathways. Two of the aforementioned enriched KEGG pathways (metabolism of xenobiotics and ribosome) were related to the well-known function and composition of the microsome (Figure 4). Enzyme Commission numbers (EC no., e.g, 1.14.14.1) are used to represent enzymes in metabolism. Highlighted in green background are known mouse enzymes annotated in the KEGG database and the red boxed are enzymes in our dataset (Figure 4(a)). All enzymes (*n* = 9) that played a key role in every pathway of metabolism of xenobiotics were included in our dataset (Table 4). Thirteen proteins from large and small subunits of ribosome were also found in our dataset (Table 4) and are indicated with a red box (Figure 4(b)). These proteins interact physically with each other and form a large protein complex—the ribosome. All the identified proteins that are involved in those two pathways are summarized in Table 4, including their KEGG pathway, protein ID, and protein name.

## 4. Discussion

Proteome analysis of the cell membrane-bound organelles is a daunting task mainly because of (a) isolation of membrane that is free from nonconstituents and (b) solubilization of membrane proteins in a manner amenable to isoelectric focusing [10]. 2DE is an effective tool to survey biological complexity at the molecular level and provides a systematic and comprehensive study of the proteins. However, because of the *PI* value range limited by the IPG strip and the high dependence on sample preparation, some problems exist for the available 2DE protocols to resolve membrane-associated proteins [10, 22]. Therefore, in the current study, the whole microsome lysate was arrayed with 2DE, and the membrane fraction of microsomes purified by the carbonate procedure was separated with 1DE. The complementary 2DE and 1DE approaches provided a much wider coverage of microsome proteome. 

Hydrophobicity and relatively low abundance causes a challenge for proteomic technology to separate and identify membrane proteins. The hydrophobicity of proteins is frequently expressed as GRAVY scores (http://us.expasy.org/tools/protparam.html). A calculated GRAVY score of up to –0.4 indicates a hydrophobic protein, suggesting a membrane association [21]. In the current study, 69 (70%) of the 99 proteins identified from 1DE had a GRAVY > −0.4 (Supplemental Figure  3), indicating the probability for membrane association [21]. As shown in Supplemental Figure  4, some alkaline proteins with *PI* values close to or greater than 10 were separated by 1DE; they could not be detected in conventional 2DE map. Only 23 proteins were found to be consistent between 2DE and 1DE datasets with 6 proteins classified as membrane proteins (Table 3). All these results indicate that 1DE is a potent supplement to 2DE, and the combination of the two approaches is necessary in protein profiling of microsomes.

Microsome-sealed vesicles could be converted into flat membrane sheets with cisternal contents that were released effectively with the treatment solution (100 mM Na_2_CO_3_; 0°C). It appears to be as effective as the low detergent procedure in selectively releasing microsomal content. In the current study, some proteins that were identified from Na_2_CO_3_-extracted fraction were classified as membrane associated mainly based on published reports, even though their predicted transmembrane domains (TMDs) did not suggest a membrane origin. The observations point out the fact that structure alone may not be the deciding factor, as far as the association of proteins with cell membrane is concerned. First, the proteins may be bound to the membrane simply to perform their functional obligations. Consequently, they could become part of complexes involving membrane proteins and may not depart from them easily under the conditions of sample preparation. For example, many enzymes were identified in the extracted membrane fraction, such as Cis-retinol androgen dehydrogenase 1 (short-chain dehydrogenase family). It is anchored to the ER membrane facing the cytoplasm by an N-terminal signaling sequence of 22 residues and takes part in the membrane-associated retinoid metabolism [23], so is fatty acid-binding protein, which participates in the palmitic acid or retinylester metabolism that is incorporated in microsomal membranes [24] and the free fatty acid transferation to the membrane. Second, some truly cytosolic proteins may simply integrate with membrane vesicles during the sonication process and become difficult to remove by the extraction methods [25]. Studies [5] have demonstrated that hepatic microsomes are derived from the ER and other cell organelles. The ER represents a membrane tubular network that crosses the cytoplasm from the nucleus membrane to the plasma membrane. Moreover, some proteins perform their functions between cytoplasm and ER, such as fatty-acid-binding proteins [26]. From this point of view, taking all of the portions into account, 60%–70% of the proteins identified can be regarded as microsome proteins in this research. A part (~15%) of identified proteins did not have unambiguous locations in published reports or annotations in the genome database. This current study provides information relevant to subcellular locations of these proteins for subsequent studies.

Two datasets from 1DE and 2DE are part of the complete protein composition of microsomes. A bioinformatics analysis of the two datasets combined offers more information. For an overview of the proteomic data and comprehending their biological importance, biological networks GO (BinGO) (http://www.psb.ugent.be/cbd/papers/BiNGO/index.html) was used to identify GO-category significant enrichment with all the identified proteins. BiNGO is a plugin for Cytoscape, which is an open source bioinformatics software platform to visualize and integrate molecular interaction networks. BinGO maps the predominant functional themes of a given gene set on the GO hierarchy. Of the 259 target proteins and direct partners analyzed, 182 target proteins linked to one or more GO terms. GO-term enrichment analysis revealed that the most highly represented GO terms in the cellular GO category component were organelles such as ER, mitochondrial, and organelle membrane. An analysis of the proteins that were identified according to their potential roles in biological processes indicated that the proteins were mainly involved in metabolic process, localization, transport, and translation. All the results were highly statistically significant.

The KEGG pathway database integrates current knowledge on molecular interaction networks in biological processes. To gain a broad understanding of our dataset, WebGestalt (a web-based gene set analysis toolkit) was used to map the identified proteins to KEGG pathways. The results showed that 112 of the total proteins were associated with one or more KEGG pathways. Meanwhile, 97 of 112 target proteins (87%) fell into 34 KEGG pathways; they were specifically enriched (*P* < 0.01) compared to statistical expectations. Pathways that are involved in benzoate degradation, metabolism of xenobiotic, glutamate metabolism, and cysteine metabolism were among the most enriched biologically. This finding was consistent with the fact that microsomes were used to investigate the metabolism of compounds and to examine drug-drug interaction by in vitro studies.

Collectively, the bioinformatics analysis via enrichment analysis of GO annotation and KEGG pathways derived meaning from the proteomic data and assisted in the understanding of the function of liver at the subcellular level.

**Novelty and Limitation**

Mammalian liver microsome proteomes have been studied by several groups [18–20]. Comparison of the current study with the literature data [18–20] was shown in Tables 5 and 6. Zgoda et al. [18] studied differential ultracentrifugation-separated mouse liver microsome proteome; 2DE and silver stain yielded 1,100 protein spots, and 138 proteins contained in 2D gel spots were identified with peptide mass fingerprint (PMF). Zgoda et al. [19] also studied differential ultracentrifugation-separated mouse liver microsome proteome with 1DE and MS/MS; 519 proteins were identified including 138 (138/519 = 27%) predicted membrane proteins. Gilchrist et al. [20] used 1DE and MS/MS to analyze rat ER and Golgi that were separated with differential ultracentrifugation and density gradient centrifugation; 832 ER proteins were identified including 183 (183/832 = 22%) membrane proteins. This current study combined differential ultracentrifugation and sucrose gradient centrifugation to prepare mouse liver microsomes; 2DE and Coomassie brilliant blue stain yielded 514 protein spots, and 183 proteins were identified with MS/MS from 204 excised gel spots, including 41 (41/183 = 22%) membrane proteins. Na_2_CO_3_ was used to further extract membrane proteins from isolated microsomes; 1DE and Coomassie brilliant blue stain yield 17 protein bands, and 99 proteins were identified with MS/MS from those 17 protein bands, including 54 (54/99 = 55%) membrane proteins. A total of 259 nonredundant proteins were identified including 62 (62/259 = 24%) membrane proteins. Compared to the documented data [18–20], the novelty of this current study is that the carbonate method significantly increased the identification rate of microsomal membrane proteins, that some proteins and functional annotations from this current study have not been identified in other literature, which expanded and enriched the documented data, and that the established analysis system and data will benefit the discovery of liver disease-related microsomal membrane proteins. Meanwhile, we also noted that the current study had a relatively low coverage (*n* = 259 proteins) of mouse liver microsome proteome relative to the documented data (*n* = 519 proteins [19] and 832 proteins [20]), which might be derived from several factors: (i) inconsistent protein-extracted procedures and protein-stained methods were used, (ii) only part of 2D gel spots were excised to identify proteins, (iii) only visualized 1D gel bands (not the entire 1D gel lane) were used for protein identification, (iv) MS/MS (not PMF) was used to identify 2D gel proteins, (v) different sensitivity mass spectrometers were used, (vi) different parameters were used to search protein database. The use of 2D/3D LC-MS/MS [19] and carbonate extraction of isolated microsomes would significantly improve the coverage of microsomal membrane proteome.

## 5. Conclusions

The preparation of liver microsomes was optimized. The data presented here demonstrated that 1DE and 2DE are complementary approaches to analyze the intracellular microsomes that contain considerable numbers of highly hydrophobic membrane proteins. An integrated bioinformatics analysis of all of the microsome proteins identified with 1DE and 2DE can provide a relatively complete understanding of the protein composition and cellular function of the target microsome organelles. The information presented here will be useful for successful analysis of other membranous organelles. Our data will assist in understanding the function of liver and are an important reference for subsequent analysis of liver disease-related microsome proteins for biomarker discovery and mechanism clarification of a liver disease.

## Supplementary Material

Supplemental figure 1: is detection and validation of the purity of isolated microsomes.
Supplemental figure 2: is distribution of subcellular locations of 2DE-derived proteins.
Supplemental figure 3: is distribution of 1DE-derived proteins over the GRAVY scores.
Supplemental figure 4: is distribution of 1DE-derived proteins over the pI values.
Supplemental figure 5: is significant enrichment of GO terms for mouse liver microsome proteins *(n* = 259) that were derived from 1DE and 2DE strategies.
Supplemental Table 1: MS/MS identification of 2DE-arrayed proteins from mouse liver microsomal preparations.
Supplemental Table 2: MS/MS identification of 1DE-separated proteins from Na_2_CO_3_-extracted mouse liver microsomal membrane preparations

## Figures and Tables

**Table 1:** Proteins identified from mouse liver microsomal preparations with 2DE-based strategy.

Spot no.	IPI^a^	Protein name	Predicted TMD	Location
90,91	IPI00108939	glyceraldehyde-3-phosphate dehydrogenase, spermatogenic	0	ER
6	IPI00111936	UDP-glucuronosyltransferase 1-2 precursor, microsomal	1	ER
145	IPI00121833	Acetyl-coenzyme A acyltransferase 1	0	ER
102	IPI00622235	Transitional endoplasmic reticulum ATPase	0	ER
6,61,194	IPI00122815	Prolyl 4-hydroxylase, beta polypeptide	0	ER
17	IPI00123176	Similar to glyceraldehyde-3-phosphate dehydrogenase, 37 kDa protein	0	ER
134,135	IPI00123342	Hypoxia upregulated 1	1	ER
2	IPI00129526	Endoplasmin	0	ER
139	IPI00131459	Nucleoside diphosphate kinase A	0	ER
179	IPI00132874	Splice isoform 1 of monoglyceride lipase	0	ER
163	IPI00133522	Protein disulfide-isomerase precursor	0	ER
49	IPI00134058	Thioredoxin domain containing protein 4 precursor	0	ER
108,145,65	IPI00135284	Similar to glyceraldehyde-3-phosphate dehydrogenase (GAPDH) ISOFORM 1	0	ER
147,148,149	IPI00135686	Mus musculus adult male kidney cDNA, RIKEN full-length enriched library, clone: 0610008	1	ER
174,178,179,183	IPI00135726	Similar to glyceraldehyde-3-phosphate dehydrogenase (GAPDH)	0	ER
49,50	IPI00163011	Thioredoxin domain containing protein 5 precursor	0	ER
137	IPI00226993	Thioredoxin	0	ER
148	IPI00229551	ADAM4	1	ER
62,157,158,162	IPI00230108	Glucose-regulated protein, full insert sequence	0	ER
148	IPI00271869	Similar to glyceraldehyde-3-phosphate dehydrogenase (GAPDH)	0	ER
146,147,149,150,153	IPI00273646	Glyceraldehyde-3-phosphate dehydrogenase	0	ER
187	IPI00555023	Glutathione S-transferase P 1	0	ER
144	IPI00319652	Glutathione peroxidase	0	ER
84	IPI00319992	78 kDa glucose-regulated protein precursor	0	ER
153	IPI00320208	Elongation factor 1-beta	0	ER
118	IPI00323357	Heat shock cognate 71 kDa protein	0	ER
173	IPI00323661	Similar to glyceraldehyde-3-phosphate dehydrogenase (GAPDH)	0	ER
145,201	IPI00462605	Similar to glyceraldehyde-3-phosphate dehydrogenase (GAPDH)	0	ER
127	IPI00469307	Alpha-2-macroglobulin receptor-associated protein precursor	0	ER
143,152,203	IPI00480343	2700050F09Rik protein	0	ER
162	IPI00831714	Leucine-rich repeat-containing protein 7	0	ER (integral to membrane)
149,150	IPI00352124	Flavin containing monooxygenase 5	1	ER (integral to membrane)
131	IPI00132397	GTP-binding protein SAR1b	0	ER (peripheral membrane protein)
107	IPI00227657	Stromal cell-derived factor 2-like protein 1 precursor	0	ER lumen
145	IPI00123281	Expressed sequence AA959742	1	ER membrane
7	IPI00222496	Protein disulfide isomerase-associated 6	1	ER, membrane protein^b^
7	IPI00112322	UDP-glucuronosyltransferase 2B5 precursor	1	ER, membrane proteins^b^
156	IPI00331322	Microsomal glutathione S-transferase 1	3	ER, outer membrane
151	IPI00319973	Membrane-associated progesterone receptor component 1	1	ER, membrane-bound
152	IPI00170316	Multiple coagulation factor deficiency protein 2 homolog precursor	0	ER-Golgi intermediate compartment
170	IPI00408892	RAS-related protein RAB-7	0	Golgi, endosomes, lysosomes
153	IPI00123316	Splice isoform 1 of tropomyosin 1 alpha chain	0	Cytoplasm
116	IPI00133456	Senescence marker protein-30	0	Cytoplasm
89,129,130,151,152	IPI00135085	Heme-binding protein	0	Cytoplasm
165	IPI00109061	Tubulin beta-4 chain homolog	0	Cytoplasmic
88	IPI00109073	Tubulin beta-4 chain	0	Cytoplasmic
105,138	IPI00110753	Tubulin alpha-1 chain	0	Cytoplasmic
113,166,167,197,204	IPI00110827	Actin, alpha skeletal muscle	0	Cytoplasmic
9,129,130,151.153,166, 167,197,198	IPI00110850	Actin, cytoplasmic 1	0	Cytoplasmic
90	IPI00114162	Fatty acid-binding protein, epidermal	0	Cytoplasmic
145	IPI00116277	T-complex protein 1, delta subunit	0	Cytoplasmic
144	IPI00117264	DJ-1 protein	0	Cytoplasmic
191,164,165	IPI00117348	Tubulin alpha-2 chain	0	Cytoplasmic
137,138,164	IPI00117350	Tubulin alpha-4 chain	0	Cytoplasmic
141,153,165,132	IPI00117352	Tubulin beta-5 chain	0	Cytoplasmic
126	IPI00117914	Arginase 1	0	Cytoplasmic
152	IPI00120532	21 kDa protein	0	Cytoplasmic
107,108,139,143,182	IPI00125489	44 KD protein (Argininosuccinate synthase)	0	Cytoplasmic
191	IPI00626790	Glutamine synthetase	0	Cytoplasmic
176,182,194	IPI00130950	Betaine-homocysteine S-methyltransferase	0	Cytoplasmic
99	IPI00131204	UDP-glucose pyrophosphorylase 2	0	Cytoplasmic
204	IPI00136929	Gamma actin-like protein	0	Cytoplasmic
101,132	IPI00169463	Tubulin beta-2C Chain	0	Cytoplasmic
202,133	IPI00221400	Alcohol dehydrogenase A chain	0	Cytoplasmic
89	IPI00221528	Actin, cytoplasmic type 5 homolog	0	Cytoplasmic
168	IPI00221890	Carbonic anhydrase III	0	Cytoplasmic
202,133	IPI00317740	Guanine nucleotide-binding protein beta subunit 2-like 1	0	Cytoplasmic
159	IPI00331174	T-complex protein 1, eta subunit	0	Cytoplasmic
154	IPI00338039	Tubulin, beta 2	0	Cytoplasmic
141	IPI00348094	Predicted: similar to tubulin M beta 1	0	Cytoplasmic
136	IPI00404011	Microtubule-associated protein	0	Cytoplasmic
153	IPI00421223	Tropomyosin alpha 4 chain	0	Cytoplasmic
194,195	IPI00457825	Similar to argininosuccinate synthase (Citrulline-aspartate ligase)	0	Cytoplasmic
60	IPI00462072	Similar to alpha enolase (2-phospho-D-glycerate hydro-lyase)	0	Cytoplasmic
178	IPI00467066	Glycine N-methyltransferase	0	Cytoplasmic
63,109	IPI00467833	Triosephosphate isomerase	0	Cytoplasmic
153	IPI00605380	Similar to tubulin alpha-2 chain (Alpha-tubulin 2)	0	Cytoplasmic
162	IPI00123313	Ubiquitin-activating enzyme E1 1	0	Cytoplasmic and nuclear
64	IPI00420745	Proteasome subunit, alpha type 2, full insert sequence	0	Cytoplasmic and nuclear
145	IPI00320165	Oxidoreductase HTATIP2	0	Cytoplasmic and nuclear
153	IPI00117978	Cytochrome c oxidase subunit IV isoform 1, mitochondrial precursor	1	Mitochondrial inner membrane
19	IPI00109167	NADH-ubiquinone oxidoreductase 24 kDa subunit	0	Mitochondrial inner membrane
158	IPI00111885	Ubiquinol-cytochrome-c reductase complex core protein I, mitochondrial precursor	0	Mitchondrial inner membrane
175	IPI00121322	Electron transfer flavoprotein-ubiquinone oxidoreductase, mitochondrial precursor	0	Mitchondrial inner membrane
196	IPI00128023	NADH-ubiquinone oxidoreductase 49 kDa subunit, mitochondrial precursor	0	Mitchondrial inner membrane
134	IPI00111908	Predicted: carbamoyl-phosphate synthetase 1	0	Mitochondrial
145	IPI00114840	Endonuclease G, mitochondrial precursor	0	Mitochondrial
70	IPI00331555	2-oxoisovalerate dehydrogenase alpha subunit, mitochondrial precursor	0	Mitochondrial
94,95	IPI00115607	Trifunctional enzyme beta subunit, mitochondrial precursor	0	Mitochondrial
145	IPI00115824	NipSnap1 protein	0	Mitochondrial
22	IPI00116154	Cytochrome c oxidase, subunit vb, full insert sequence	0	Mitochondrial
15,146,147,148,149,100	IPI00118986	ATP synthase oligomycin sensitivity conferral protein, mitochondrial precursor	0	Mitochondrial
127	IPI00119138	Ubiquinol-cytochrome-c reductase complex core protein 2, mitochondrial precursor	0	Mitochondrial
147,148	IPI00120984	NADH-ubiquinone oxidoreductase 19 kDa subunit	0	Mitochondrial
137	IPI00129516	Ubiquinol-cytochrome c reductase complex 11 kDa protein, mitochondrial precursor	0	Mitochondrial
93,99,100,192,203	IPI00130280	ATP synthase alpha chain, mitochondrial precursor	0	Mitochondrial
149,150	IPI00132217	Tetratricopeptide repeat protein 11	1	Mitochondrial
150,151	IPI00132390	NADH-ubiquinone oxidoreductase B15 subunit	1	Mitochondrial
101,132,137,141,153	IPI00170093	NADH-ubiquinone oxidoreductase 23 kDa subunit, mitochondrial precursor	0	Mitochondrial
92,93,94,95,96	IPI00223092	Hydroxyacyl-coenzyme A dehydrogenase/3-ketoacyl-coenzyme A	0	Mitochondrial
142,143,152	IPI00230507	ATP synthase D chain, mitochondrial	0	Mitochondrial
162	IPI00308882	NADH-ubiquinone oxidoreductase 75 kDa subunit, mitochondrial precursor	0	Mitochondrial
149	IPI00344004	13 KDa differentiation-associated protein	0	Mitochondrial
145,146	IPI00420718	Hydroxymethylglutaryl-CoA synthase, mitochondrial precursor	0	Mitochondrial
51	IPI00308885	60 kDa heat shock protein, mitochondrial	0	Mitochondrial
153	IPI00462250	Similar to adenine nucleotide translocase	3	Mitochondrial
85,165,167,203	IPI00468481	ATP synthase beta chain, mitochondrial precursor	0	Mitochondrial
147	IPI00117281	Phospholipid hydroperoxide glutathione peroxidase, mitochondrial precursor	0	Mitochondrial and cytoplasmic
169	IPI00133240	Ubiquinol-cytochrome c reductase iron-sulfur subunit, mitochondrial precursor	0	Mitochondrial inner membrane
200,201	IPI00230351	Succinate dehydrogenase [ubiquinone] flavoprotein subunit, mitochondrial precursor	0	Mitochondrial inner membrane
174	IPI00132042	Pyruvate dehydrogenase E1 component beta subunit, mitochondrial precursor	0	Mitochondrial matrix
156	IPI00315794	Cytochrome b5 outer mitochondrial membrane isoform precursor	1	Mitochondrial outer membrane
65,109,144,170,176,100	IPI00134746	Argininosuccinate synthase	0	mitochondrion
145,146	IPI00338536	Succinate dehydrogenase [ubiquinone] iron-sulfur protein, mitochondrial precursor	0	Mitochondrion
112	IPI00122547	Voltage-dependent anion-selective channel protein 2	0	Mitochondrion outer membrane
147	IPI00131186	Splice isoform 2 of transcription factor BTF3	0	Nuclear
149	IPI00317794	Nucleolin	0	Nuclear
53	IPI00331146	UMP-CMP kinase	0	Nuclear
150	IPI00458856	Similar to ZNF91L isoform 1	0	Nuclear
6	IPI00461822	E1A binding protein p300	0	Nuclear
55	IPI00126172	RIKEN cDNA 4931406C07, PTD012 homolog	0	Nuclear
150,151	IPI00113241	40S ribosomal protein S19	0	Ribosome
104	IPI00116908	Similar to 40 s ribosomal protein S12	0	Ribosome
147	IPI00849793	60S ribosomal protein L12	0	Ribosome
81,127,145	IPI00125971	26S protease regulatory subunit S10B	0	Ribosome
199	IPI00123604	40S ribosomal protein SA	0	Ribosome
125	IPI00135640	26S protease regulatory subunit 8	0	Ribosome
149,150,151	IPI00139780	60S ribosomal protein L23	0	Ribosome
149,150	IPI00222546	60S ribosomal protein L22	0	Ribosome
145	IPI00314950	60S acidic ribosomal protein P0	0	Ribosome
149	IPI00322562	40S ribosomal protein S14	0	Ribosome
146	IPI00331092	40S ribosomal protein S4, *X* isoform	0	Ribosome
149,150	IPI00331461	60S ribosomal protein L11	0	Ribosome
188	IPI00351894	Similar to ribosomal protein	0	Ribosome
148	IPI00849793	60S ribosomal protein L12	0	Ribosome
149	IPI00465880	40S ribosomal protein S17	0	Ribosome
199	IPI00123604	40S ribosomal protein SA	0	Ribosome
4,11	IPI00121209	Apolipoprotein A-I precursor	0	Secreted
149	IPI00121837	Ribonuclease 4 precursor	1	Secreted
12	IPI00122429	Plasma retinol-binding protein precursor	0	Secreted
163	IPI00123920	Alpha-1-antitrypsin 1–3 precursor	0	Secreted
163	IPI00123924	Alpha-1-antitrypsin 1–4 precursor	0	Secreted
163	IPI00123927	Alpha-1-antitrypsin 1–5 precursor	0	Secreted
162	IPI00128484	Hemopexin precursor	1	Secreted
3,117,118	IPI00131695	Serum albumin precursor	0	Secreted
98	IPI00139788	Serotransferrin precursor	0	Secreted
199	IPI00323571	Apolipoprotein E precursor	0	Secreted
135,136	IPI00377351	Apolipoprotein A-IV precursor	0	Secreted
163	IPI00406302	Alpha-1-antitrypsin 1-1 precursor	0	Secreted
100,155,156	IPI00466399	21 kDa protein	0	Secreted
156	IPI00480401	Major urinary protein 1 precursor	0	Secreted
122	IPI00130661	Tripeptidyl-peptidase I precursor	0	Secreted (lysosomal)
101	IPI00115302	Branched chain ketoacid dehydrogenase E1, beta polypeptide	0	Membrane
197	IPI00120716	Guanine nucleotide-binding protein G(I)/G(S)/G(T) beta subunit 1	0	Membrane
21,137	IPI00120719	Cytochrome c oxidase, subunit va, full insert sequence	0	Membrane
125	IPI00124790	Polyposis locus protein 1-like 1	3	Membrane
129	IPI00132076	Catechol O-methyltransferase	1	Membrane
130,142	IPI00138406	Ras-related protein Rap-1A	0	Membrane
174	IPI00162780	Guanine nucleotide-binding protein G(I)/G(S)/G(T) beta subunit 2	0	Membrane
88,154	IPI00230113	Cytochrome b5	1	Membrane
199,200	IPI00353727	Annexin A4	0	Membrane
110	IPI00117416	Neighbor of COX4	0	Unknown
143	IPI00121271	Hypothetical S-adenosyl-L-methionine-dependent methyltransferases structure containing protein	0	Unknown
144,108	IPI00267667	RIKEN cDNA 6330409N04, CLLL6 protein homolog	0	Unknown
101	IPI00269613	Eukaryotic translation initiation factor 3 subunit 2	0	Unknown
149,150	IPI00307837	51 kDa protein	0	Unknown
203	IPI00318204	Sid6061p	0	Unknown
105	IPI00273646	Similar to glyceraldehyde-3-phosphate dehydrogenase	0	Unknown
189,190,194	IPI00626790	Glutamine synthetase	0	Unknown
50	IPI00345842	86 KDa PROTEIN	0	Unknown
51	IPI00350780	45 kDa protein	0	Unknown
133	IPI00381231	77 KDa protein	0	Unknown
144	IPI00923085	Probable ubiquitin-conjugating enzyme E2 FLJ25076 homolog	0	Unknown
146,147,150,173	IPI00460295	44 KDa protein	0	Unknown
156	IPI00330913	Major urinary protein 26	0	Unknown
59	IPI00467988	169 kDa protein	0	Unknown
100,155,156	IPI00469517	21 kDa protein	0	Unknown
149	IPI00130554	Splice isoform 1 of SNARE-associated protein Snapin	0	Vesicular membrane
101,127,134	IPI00131366	Keratin, type II cytoskeletal 6B	0	Sarcolemma
83,106,107	IPI00121788	Peroxiredoxin 1	0	Microbody
101,139	IPI00348328	Keratin Kb40	0	Intermediate filament
156	IPI00137414	Left-right dynein	0	Cilium

^a^ESI-Q-TOF identification, subcellular location are given for each ID number.

^b^This protein is nonmembrane associated according to the annotation in the Swiss-Prot database but has one predicted TMD.

**Table 2:** Proteins identified from Na_2_CO_3_-extracted mouse liver microsomal membrane preparations with 1DE-based strategy.

Bands no.	Accession no.	Protein name	Predicted TMD	GRAVY score	*PI* value	Subcellular location
9	IPI00112322	UDP-glucuronosyltransferase 2B5 precursor	1	−0.031	7.94	ER
9	IPI00127223	UDP glucuronosyltransferase 2 family, polypeptide B36	1	−0.036	8.47	ER
9	IPI00222496	Protein disulfide-isomerase A6	1	−0.292	5.05	ER
8	IPI00417182	UDP-glycosyltransferase 1 family polypeptide A5	1	0.044	8.33	ER
9	IPI00116572	Cytochrome P450, family 2, subfamily d, polypeptide 9	2	−0.043	6.37	ER
15	IPI00113655	40S ribosomal protein S6	0	−0.918	10.68	ER
5	IPI00129526	Endoplasmin precursor (ER protein 99,94 kDa glucose-regulated protein)	0	−0.72	4.74	ER
13	IPI00130985	Short-chain dehydrogenase CRAD2	0	0.026	8.35	ER
6	IPI00222809	Similar to GDH/6PGL endoplasmic bifunctional protein	0	−0.18	6.61	ER
8	IPI00230108	Glucose-regulated protein, full insert sequence	0	−0.479	5.78	ER
10,11	IPI00317356	Paraoxonase 1	0	−0.01	5.02	ER
7	IPI00319992	78 kDa glucose-regulated protein precursor	0	−0.481	5.07	ER
13	IPI00121079	NADH-cytochrome b5 reductase 3	0	−0.203	8.56	ER, membrane bound
9	IPI00123964	Cytochrome P450 2A5	1	−0.203	9.23	ER, membrane bound
9	IPI00114779	Cytochrome P450 2C38	0	−0.147	8.69	ER, membrane bound
17	IPI00331322	Microsomal glutathione S-transferase 1	3	0.14	9.67	ER and mitochondrial outer membrane
17	IPI00119766	Cis-retinol androgen dehydrogenase 1	0	0.005	9.25	ER lumen
8	IPI00134691	UDP-glucuronosyltransferase 1-1 precursor, microsomal	2	0.087	8.87	ER, integral to plasma membrane
8	IPI00128287	Cytochrome P450 1A2	1	−0.203	8.92	ER, membrane bound
10	IPI00136910	Cytochrome P450 2D11	2	−0.009	6.15	ER, membrane bound
9	IPI00308328	Cytochrome P450 2F2	1	−0.135	7.74	ER, membrane bound
9,10	IPI00323908	Cytochrome P450 2D10	2	−0.073	6.16	ER, membrane bound
7	IPI00112549	Long-chain-fatty-acid-CoA ligase 1	1	−0.045	6.81	ER, type III membrane protein
8	IPI00133522	Protein disulfide-isomerase precursor	0	−0.386	4.79	ER
9	IPI00116572	Cytochrome P450 2D9	0	−0.063	5.93	ER, membrane bound
5,6	IPI00119618	Calnexin precursor	1	−0.875	4.5	ER, type I membrane protein
1,10,12,14,15	IPI00319973	Membrane-associated progesterone receptor component 1	1	−0.616	4.57	ER, membrane bound
8	IPI00132475	Protein ERGIC-53	1	−0.545	5.92	ER-Golgi intermediate compartment (ERGIC), type I membrane protein
8,17	IPI00109061	Tubulin beta-4 chain homolog	0	−0.406	4.78	Cytoplasmic
10	IPI00110827	Actin, alpha skeletal muscle	0	−0.232	5.23	Cytoplasmic
10,12,14	IPI00110850	Actin, cytoplasmic 1	0	−0.2	5.29	Cytoplasmic
1,2,3	IPI00111908	Carbamoyl-phosphate synthase	0	−0.12	6.42	Cytoplasmic
1,8,9,13	IPI00117348	Tubulin alpha-2 chain	0	−0.23	4.94	Cytoplasmic
9,10,11,12,13	IPI00117914	Arginase 1	0	−0.187	6.52	Cytoplasmic
17	IPI00120451	Fatty acid-binding protein, liver	0	−0.409	8.59	Cytoplasmic
9	IPI00129028	Similar to tubulin, alpha 3C isoform 1	0	−0.204	4.98	Cytoplasmic
1–11,13,17	IPI00130950	Betaine-homocysteine S-methyltransferase	0	−0.36	8.01	Cytoplasmic
1,4,6,10,11,14,15	IPI00134746	Argininosuccinate synthase	0	−0.361	8.36	Cytoplasmic
3,4	IPI00114710	Pyruvate carboxylase, mitochondrial precursor	0	−0.173	6.25	Mitochondrial
17	IPI00553333	Hemoglobin subunit beta-1	0	0.092	7.13	Mitochondrial
9	IPI00134809	Dihydrolipoyllysine-residue succinyltransferase component of 2-oxoglutarate dehydrogenase complex	0	−0.171	9.1	Mitochondrial
17	IPI00117978	Cytochrome c oxidase subunit IV isoform 1, mitochondrial precursor	1	−0.412	9.25	Mitochondrial inner membrane
15,16	IPI00315794	Cytochrome b5 outer mitochondrial membrane isoform precursor	1	−0.602	4.79	Mitochondrial outer membrane
13	IPI00321718	Prohibitin-2	0	−2.58	9.83	Mitochondrial, cytoplasmic, nuclear
13	IPI00122547	Voltage-dependent anion-selective channel protein 2	0	−0.223	7.44	Outer mitochondrial Membrane
17	IPI00114559	Histone H2A type 1	0	−0.572	11.22	Nuclear
16,17	IPI00114642	Histone H2B F	0	−0.762	10.32	Nuclear
8	IPI00387318	Cell cycle control protein 50A	2	−0.331	8.58	Membrane
15	IPI00113849	Splice isoform 2 of cell division control protein 42 homolog	0	−0.157	6.16	Membrane
13	IPI00122549	Splice isoform Pl-VDAC1 of voltage-dependent anion-selective channel protein 1	0	−0.334	8.55	Membrane
15	IPI00127408	Ras-related C3 botulinum substrate 1	0	−0.101	8.77	Membrane
15	IPI00138406	Ras-related protein Rap-1A	0	−0.375	6.39	Membrane
6	IPI00116921	Scavenger receptor class B member 1	2	0.073	8.29	Integral membrane protein
1	IPI00121985	Splice Isoform 1 of solute carrier organic anion transporter family, member 1B2	12	0.172	8.95	Integral membrane protein
9	IPI00124830	Integrin-associated protein precursor	5	0.563	8.58	Integral membrane protein
14,15	IPI00131176	Cytochrome c oxidase subunit 2	2	0.27	4.6	Integral membrane protein
1	IPI00132604	Secretedretory carrier-associated membrane protein 3	4	0.028	7.55	Integral membrane protein
1	IPI00135701	Solute carrier organic anion transporter family, member 1A1	11	0.244	8.58	Integral membrane protein
1	IPI00311682	Sodium/potassium-transporting ATPase alpha-1 chain precursor	10	0.002	5.3	Integral membrane protein
6	IPI00331214	Platelet glycoprotein IV	2	−0.053	8.61	Integral membrane protein
2	IPI00119063	Prolow-density lipoprotein receptor-related protein 1	1	−0.502	5.17	Integral to membrane
1,12,16,17	IPI00124790	Polyposis locus protein 1-like 1	3	0.375	6.82	Integral to membrane
10	IPI00129677	Asialoglycoprotein receptor major subunit	1	−0.66	5.99	Integral to membrane
17	IPI00467119	Camello-like protein 1	1	0.302	9.61	Integral to membrane
5,8	IPI00316329	Keratin, type II cytoskeletal 1	0	−0.588	8.2	Intermediate filament
10,11,12	IPI00108844	Cation-dependent mannose-6-phosphate receptor precursor	1	−0.168	5.24	Type I membrane protein
9	IPI00109998	H-2 class I histocompatibility antigen, D-B alpha chain precursor	1	−0.508	6.28	Type I membrane protein
4	IPI00120245	Integrin alpha-V precursor	1	−0.246	5.46	Type I membrane protein
3,4	IPI00121190	Epidermal growth factor receptor precursor	2	−0.316	6.46	Type I membrane protein
2	IPI00126186	Macrophage mannose receptor 1 precursor	1	−0.5	6.47	Type I membrane protein
5	IPI00134549	Splice isoform LAMP-2A of lysosome-associated membrane glycoprotein 2 precursor	1	−0.036	7.05	Type I membrane protein
13	IPI00312018	Malectin	1	−0.203	5.73	Type I membrane protein
3	IPI00312063	Low-density lipoprotein receptor precursor	1	−0.391	4.88	Type I membrane protein
16	IPI00466570	Transmembrane emp24 domain-containing protein 10	2	−0.169	6.25	Type I membrane protein
4	IPI00108535	Carcinoembryonic antigen-related cell adhesion molecule 1	1	−0.302	5.35	Type I membrane protein
5	IPI00310059	Polymeric-immunoglobulin receptor precursor	1	−0.499	5.26	Type I membrane protein also secreted
9	IPI00121550	Sodium/potassium-transporting ATPase beta-1 chain	1	−0.55	8.83	Type II membrane protein
2,3	IPI00134585	Glutamyl aminopeptidase	1	−0.344	5.28	Type II membrane protein
10	IPI00307966	ADP-ribosyl cyclase 1	1	−0.106	8.64	Type II membrane protein
3	IPI00319509	Aminopeptidase N	1	−0.277	5.62	Type II membrane protein
3	IPI00458003	Ectonucleotide pyrophosphatase/phosphodiesterase 3	1	−0.346	6.13	Unknown
9	IPI00409409	CD1D1 protein	1	−0.178	9.22	Unknown
7	IPI00621548	NADPH-cytochrome P450 reductase	1	−0.463	5.37	Unknown
9	IPI00321644	Cytochrome P450 2D26	2	−0.105	6.16	Unknown
1	IPI00127016	Hydroxysteroid 17-beta dehydrogenase 6	0	−0.075	8.63	Unknown
16	IPI00221721	Hypothetical krab box containing protein, full insert sequence	0	−0.142	9.84	Unknown
8	IPI00224073	Hypothetical peptidase family M20/M25/M40 containing protein, full insert sequence	0	−0.01	5.99	Unknown
16	IPI00228379	Ferritin light chain 2	0	−0.479	6.37	Unknown
17	IPI00266842	17 kDa protein	0	−0.668	10.48	Unknown
15	IPI00379258	Similar to ferritin light chain 2	0	−0.454	8.51	Unknown
3	IPI00405742	Plexin B2	0	−0.3	5.67	Unknown
2	IPI00408258	Structure-specific endonuclease subunit SLX4	0	−0.714	5.33	Unknown
11	IPI00462251	Hypothetical protein LOC72792 isoform 1	0	−0.429	5.82	Unknown
15	IPI00605814	Similar to Ferritin light chain 1	0	−0.358	6.42	Unknown
10	IPI00131366	Keratin, type II cytoskeletal 6B	0	−0.488	8.32	Sarcolemma
10	IPI00322209	Keratin, type II cytoskeletal 8	0	−0.602	5.7	Sarcolemma
8	IPI00853991	Similar to VH coding region	0	−0.102	5.31	Secreted
10	IPI00126458	MRNA	1	−0.55	5.66	MHC class I protein complex
15	IPI00121788	Peroxiredoxin 1	0	−0.221	8.26	Microbody

**Table 3:** Proteins that are consistently present in both 2DE dataset of microsomal proteins (Table 1) and 1DE dataset of Na_2_CO_3_-extracted microsomal proteins (Table 2).

Accession number	Protein name	Predicted TMD	GRAVY scores	*PI* value	Location
IPI00108454	Similar to 40S ribosomal protein S6	0	−0.918	10.68	ER
IPI00112322^a^	UDP-glucuronosyltransferase 2B5 precursor	1	−0.031	7.94	ER
IPI00129526	Endoplasmin precursor (ER protein 99, 94 kDa glucose-regulated protein)	0	−0.72	4.74	ER
IPI00133522	Protein disulfide-isomerase precursor	0	−0.386	4.79	ER
IPI00222496^a^	Protein disulfide isomerase-associated 6	1	−0.292	5.05	ER
IPI00230108	Glucose-regulated protein, full insert sequence	0	−0.479	5.78	ER
IPI00319992	78 kDa glucose-regulated protein precursor	0	−0.481	5.07	ER
IPI00331322^a^	Microsomal glutathione S-transferase 1	3	0.14	9.67	ER and mitochondrial outer membrane
IPI00319973^a^	Membrane-associated progesterone receptor component 1	1	−0.616	4.57	ER, membrane bound
IPI00109061	Tubulin beta-4 chain homolog	0	−0.406	4.78	Cytoplasmic
IPI00110827	Actin, alpha skeletal muscle	0	−0.232	5.23	Cytoplasmic
IPI00110850	Actin, cytoplasmic 1	0	−0.2	5.29	Cytoplasmic
IPI00111908	Predicted: carbamoyl-phosphate synthetase 1	0	−0.12	6.42	Cytoplasmic
IPI00117348	Tubulin alpha-2 chain	0	−0.23	4.94	Cytoplasmic
IPI00117914	Arginase 1	0	−0.187	6.52	Cytoplasmic
IPI00134746	Argininosuccinate synthase	0	−0.361	8.36	Cytoplasmic
IPI00117978^a^	Cytochrome c oxidase subunit IV isoform 1, mitochondrial precursor	1	−0.412	9.25	Mitochondrial inner membrane
IPI00315794^a^	Cytochrome b5 outer mitochondrial membrane isoform precursor	1	−0.602	4.79	Mitochondrial outer membrane
IPI00122547^a^	Voltage-dependent anion-selective channel protein 2	0	−0.223	7.44	Outer mitochondrial membrane
IPI00124790^a^	Polyposis locus protein 1-like 1	3	0.375	6.82	Integral to membrane
IPI00138406^a^	Ras-related protein Rap-1A	0	−0.375	6.39	Membrane
IPI00121788	Peroxiredoxin 1	0	−0.221	8.26	Microbody
IPI00131366	Keratin, type II cytoskeletal 6B	0	−0.488	8.32	Sarcolemma

^a^Membrane proteins with one or more predicted trans-membrane origins or validated by references.

**Table 4:** Proteins involved in KEGG pathways. (a) Metabolism of xenobiotics. (b) Ribosome.

KEGG pathway		Protein ID	Protein name	MS-identified proteins
A. Metabolism of exnobiotics	EC:1.14.14.1	IPI00128287	Cytochrome P450 1A2	+
		IPI00123964	Cytochrome P450 2A5	+
		IPI00116572	Cytochrome P450 2D9	+
		IPI00323908	Cytochrome P450 2D10	+
		IPI00321644	Cytochrome P450 2D26	+
		IPI00114779	Cytochrome P450 2C38	+
		IPI00308328	Cytochrome P450 2F2	+
	EC:2.5.1.18	IPI00331322	Microsomal glutathione S-transferase 1	+
	EC:1.1.1.1	IPI00221400	Alcohol dehydrogenase A chain	+
B. Ribosome	Small subunit	IPI00135640	26S protease regulatory subunit 8	+
		IPI00125971	26S protease regulatory subunit S10B	+
		IPI00331092	40S ribosomal protein S4, X isoform	+
		IPI00116908	Similar to 40s ribosomal protein S12	+
		IPI00322562	40S ribosomal protein S14	+
		IPI00465880	40S ribosomal protein S17	+
		IPI00113241	40S ribosomal protein S19	+
		IPI00123604	40S ribosomal protein SA	+
		IPI00314950	60S acidic ribosomal protein P0	+
	Large subunit	IPI00331461	60S ribosomal protein L11	+
		IPI00849793	60S ribosomal protein L12	+
		IPI00222546	60S ribosomal protein L22	+
		IPI00139780	60S ribosomal protein L23	+

**Table 5:** Comparison of the current study with the literature data [18–20].

	Current study	Ref. [19]	Ref. [18]	Ref. [20]
Species	Mouse	Mouse	Mouse	Rat
Sample	Liver microsome	Liver microsome	Liver microsome	ER, Golgi
Pretreatment	None	Phenobarbital	Phenobarbital or 3-methylcholanthrene	None
Sample preparation	Subfractionated by differential ultra-centrifugation + sucrose gradient centrifugation + Na_2_CO_3_	Subfractionated by differential ultra-centrifugation	Differential ultracentrifugation	Subfractionated by differential ultra-centrifugation + density gradient centrifugation
Protein separation	2DE, 1DE	1DE	2DE	1DE
1D/2D-Gel Stain	Coomassie brilliant blue (2DE; 1DE)	—	Silver stain	—
Protein identification	MS/MS	MS/MS	PMF	MS/MS
Protein spots on 2D-Gel	514	—	1100	—
Proteins identified in 2D-Gel	183	—	139	—
Proteins identified in 1DE	99	519	—	832 (ER)
Proteins identified in 2-D LC	—	1410	—	—
Proteins identified in 3-D LC	—	3703	—	—
Total identified proteins	259	4142	Unspecified	832 (ER)
Membrane proteins	2DE: 41 (41/183 = 22%) 1DE: 54 (54/99 = 55%) Total: 62 (62/259 = 24%)	1DE: 138 (138/519 = 27%) 2-D LC: 259 (259/1410 = 21%) 3-D LC: 659 (659/3703 = 18%)	Unspecified	183 (183/832 = 22%)
Protein superfamily				
P450 family members	10	29	2	11
Ribosomal proteins	13	16	Unspecified	45
UDP glycosyltransferases, UGTs	6	8	Unspecified	3
Tubulins	11	5	Unspecified	2
Short-chain dehydrogenase/reductase	32	9	Unspecified	56
Protein disulfide isomerase	2	4	Unspecified	1

**Table 6:** Comparison of selected proteins between the current study and the literature data [18–20].

Protein	Current study	Ref. [19]	Ref. [18]	Ref. [20]
P450 family members	2D9, 2A5, 2C38, 1A2, 2D11, 2F2, 2D10, 2D26		2C37	17A1, 20A1, 2B2, 2J3, 4A1, 4A8, 4F1, 4F4, 4V3, 8B1, NA2
GRP-170	Hypoxia upregulated protein 1		170 kDa glucose regulated protein	—
Endoplasmin	Endoplasmin		Tumor rejection antigen gp96	
Serotransferrin	Serotransferrin		Transferrin	—
78 kDa glucose-regulated protein	78 kDa glucose-regulated protein		78 kDa glucose-regulated protein	—
Stress-induced phosphoprotein 1	—		Stress-induced phosphoprotein 1	—
Calreticulin family	Calnexin		Calreticulin	—
Protein disulfide-isomerase	Protein disulfide-isomerase precursor (PDI)		Protein disulfide-isomerase precursor (PDI)	Similar to disulfide isomerase
Glucose-regulated protein similar to ER-60 protease	—		Glucose-regulated protein similar to ER-60 protease	—
Erp58	—		Erp58	—
Vitamin D-binding protein	—		Vitamin D-binding protein	—
Tubulins	Tubulin beta-4, alpha-1, alpha-2, alpha-4, beta-5, beta-2C, beta 2		Tubulin alpha	Tubulin alpha 6
Fibrinogen	—		Fibrinogen, gamma polypeptide	—
Serine protease inhibitor	—		Similar to serine protease inhibitor 1–4	—
Argininosuccinate synthetase 1	Argininosuccinate synthetase 1		Argininosuccinate synthetase 1	—
Interferon-inducible GTPase	—		Interferon-inducible GTPase	
Progesterone receptor membrane component	Progesterone receptor membrane component		Progesterone receptor membrane component	—
Major urinary protein 2	Major urinary protein 2		Major urinary protein 2	—
Superoxide dismutase I	—		Superoxide dismutase I	—
Ribosomal proteins	26S protease regulatory subunit 8, S10B; 40S ribosomal protein S17, SA, S6, S19, S12, SA, S14, S4 X isoform; 60S ribosomal protein L11, L12, L23, L22, P0		Unspecified	40S Ribosomal Protein S10, S12, S18, S20, S21, S23, S24, S25, S26, S27, S29, S30, S6, S9 60S Ribosomal Protein L12, L15, L18A, L19, L21, L22, L23, L23A, L24, L26, L27, L27A, L28, L3, L32, L34, L35, L35A, L36, L37, L37A, L39, L4, L40, L44, L6, L7A
UDP glycosyltransferases, UGTs	UDP-glucuronosyltransferase 2B5, 2B36, 1A5		Unspecified	UDP-Glucuronosyltransferase 1A7
	UDP-glucuronosyltransferase 1-1 precursor			UDP-Glucuronosyltransferase GTNA2
	UDP-glucuronosyltransferase 1-2 precursor			
Short-chain dehydrogenase/reductase	Glyceraldehyde-3-phosphate dehydrogenase Alcohol dehydrogenase A Short-chain dehydrogenase CRAD2 Cis-retinol androgen dehydrogenase 1 Hydroxysteroid 17-beta dehydrogenase 6		Unspecified	Glyceraldehyde 3-phosphate dehydrogenase Alcohol dehydrogenase [NADP+] Similar to retinal short-chain Dehydrogenase/reductase Retinol dehydrogenase 10 Hydroxysteroid (17-Beta) dehydrogenase 8
	Oxidoreductase HTATIP2 NADH-ubiquinone oxidoreductase 24 kDa subunit NADH-cytochrome b5 reductase 3 NADPH-cytochrome P450 reductase		Unspecified	Oxidoreductase ero1-L endoplasmic oxidoreductase 1 Beta

No protein list was obtained from [19].—means “not included.”

## Abbreviations

ACN: Acetonitrile
BinGO: Biological Networks Gene Ontology
DTT: Dithiothreitol
1DE: One-dimensional gel electrophoresis
2DE: Two-dimensional gel electrophoresis
ER: Endoplasmic reticulum
GO: Gene ontology
GRAVY: Grand average of hydropathy
HLPP: Human Liver Proteome Project
IEF: Isoelectric focusing
KEGG: Kyoto Encyclopedia of Genes and Genomes
LC: Liquid chromatography
MS: Mass spectrometry
MS/MS: Tandem mass spectrometry
Q-TOF: Quadrupole-time of flight
RP: Reverse phase
TMD: Transmembrane domains.
