# Supplementary material for: Proteomic and Bioinformatics Analyses of Mouse Liver Microsomes
Source: Int J Proteomics. 2012 Mar 20;2012:832569. doi: 10.1155/2012/832569 (PMC3317213; doi:10.1155/2012/832569)
Supplement: Supplementary file 1 — Supplemental figure 1: is detection and validation of the purity of isolated microsomes. Supplemental figure 2: is distribution of subcellular locations of 2DE-derived proteins. Supplemental figure 3: is distribution of 1DE-derived proteins over the GRAVY scores. Supplemental figure 4: is distribution of 1DE-derived proteins over the pI values. Supplemental figure 5: is significant enrichment of GO terms for mouse liver microsome proteins (n = 259) that were derived from 1DE and 2DE strategies. Supplemental Table 1: MS/MS identification of 2DE-arrayed proteins from mouse liver microsomal preparations. Supplemental Table 2: MS/MS identification of 1DE-separated proteins from Na2CO3-extracted mouse liver microsomal membrane preparations [file 832569.f1.pdf]

Supplemental Figures

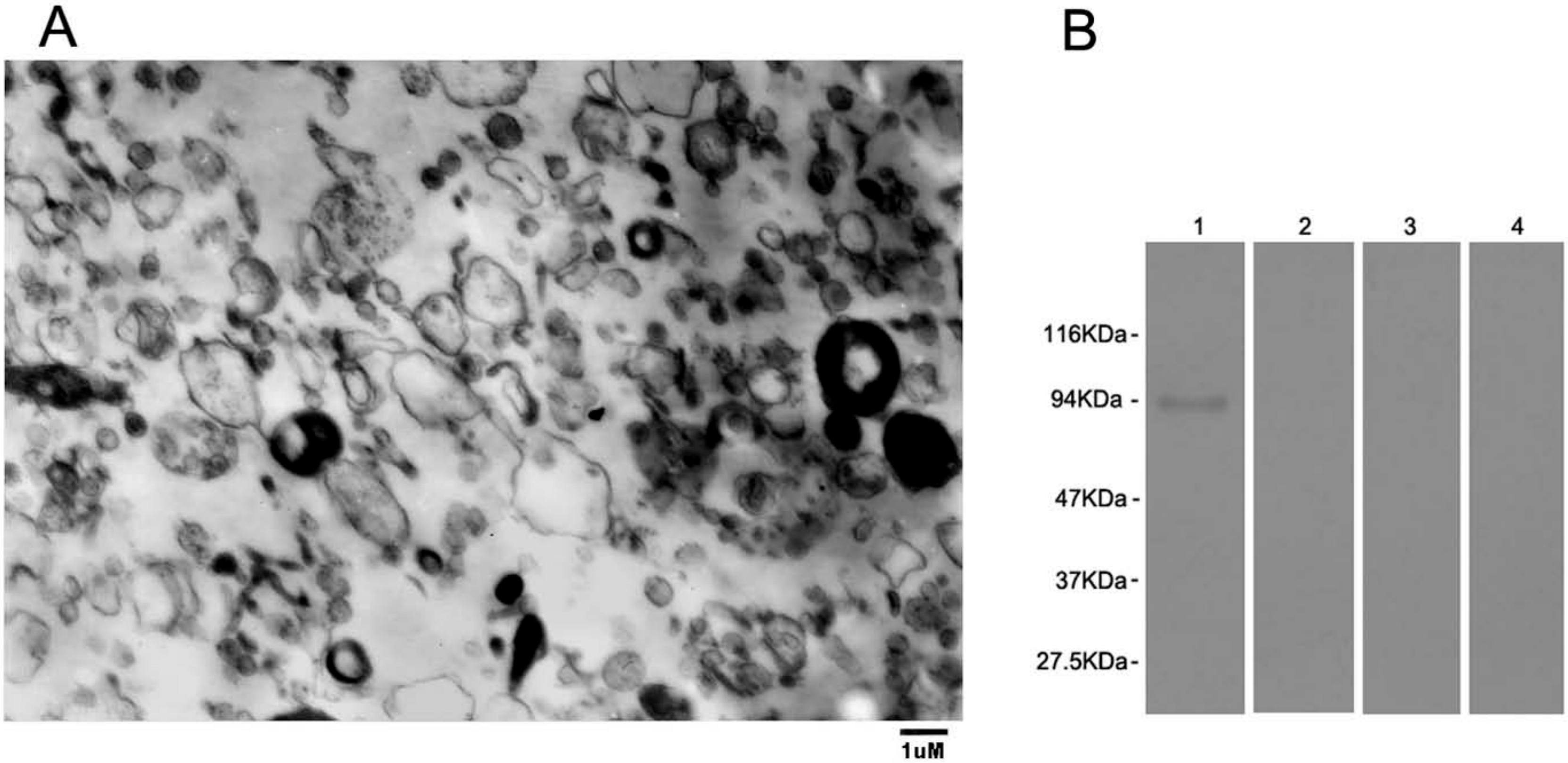

Supplemental Figure 1. Detection and validation of the purity of isolated microsomes. A. Electron microscope image. A representative area of ER vesicles was shown without apparent other apparatus contamination, observed with electron microscope. B. Western blotting image. A amount (50 µg) of proteins were loaded at each lane, and lanes 1, 2, 3 and 4 were sequentially detected with anti-endoplasmin (92.5 kDa; ER marker), anti-OxPhos Complex IV subunit I (56.9 kDa; mitochondrial marker), anti-Catalase (59.8 kDa; peroxisomal marker), and anti-cadherin (98.3 kDa; cytoplasmic marker), respectively.

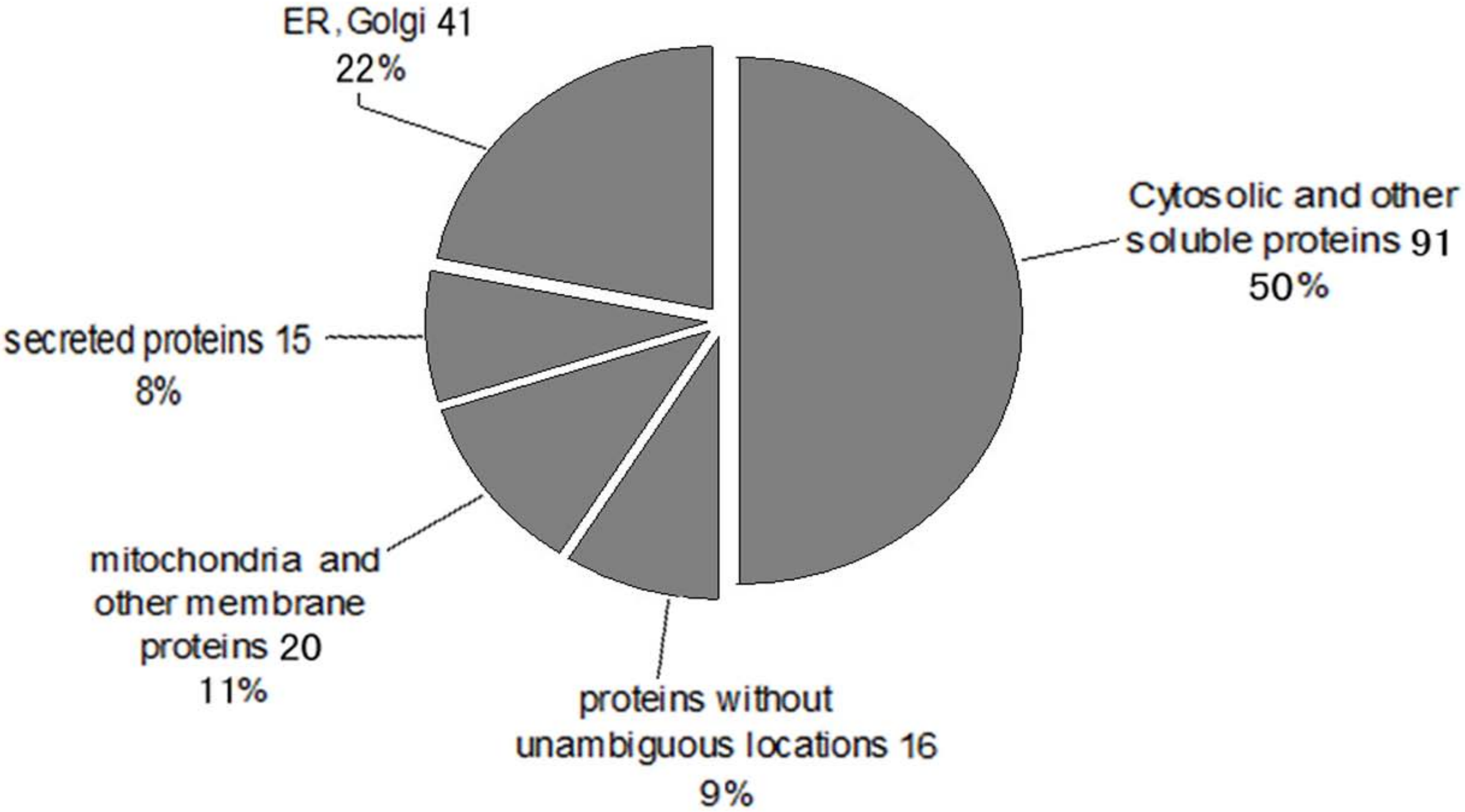

Supplemental Figure 2. **Distribution of subcellular locations of 2DE-derived proteins.** The subcellular locations were derived from Swiss-Prot Dadabase and the Gene Ontology.

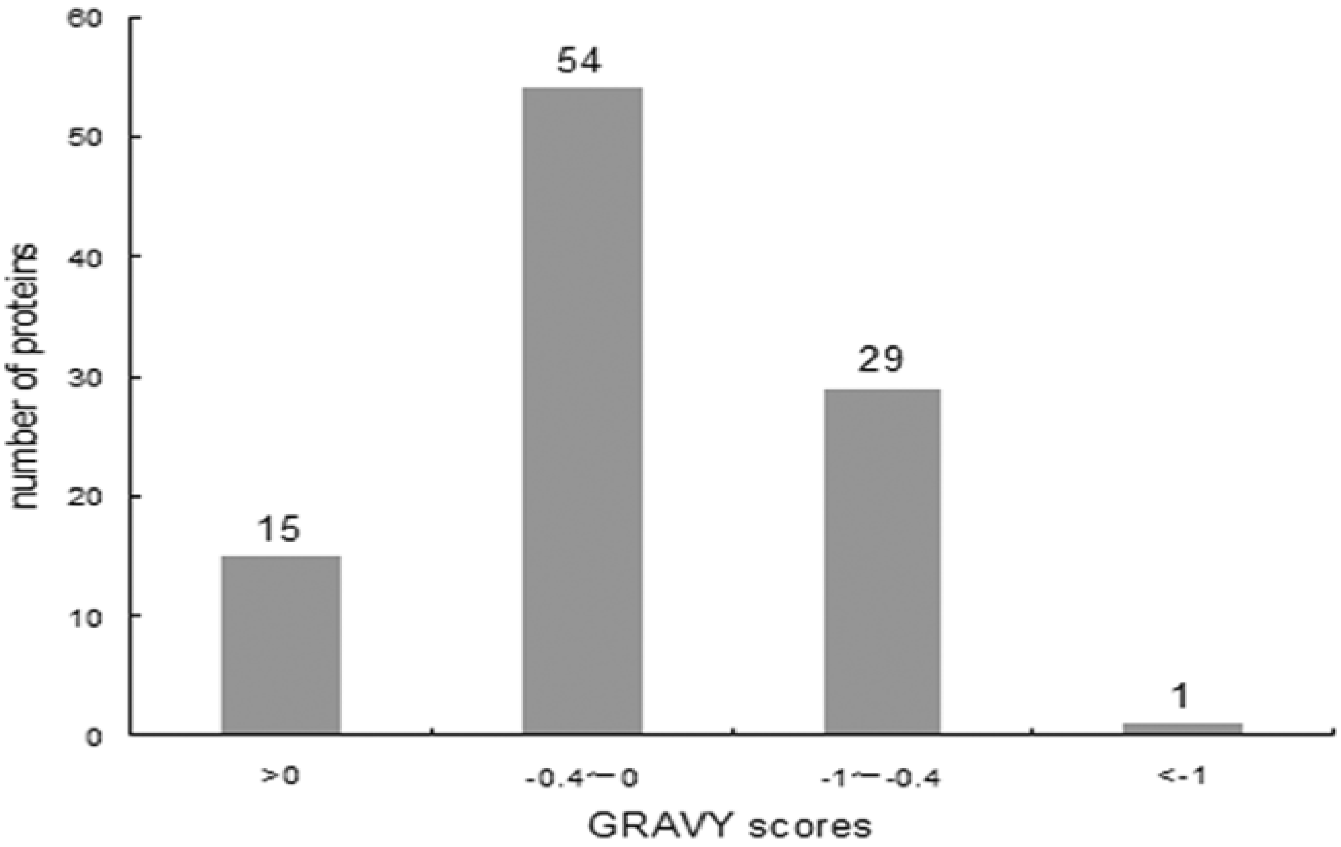

Supplemental Figure 3. Distribution of 1DE-derived proteins over the GRAVY scores. The value (-0.4) is the mean GRAVY score for cytosolic proteins; it is the borderline between cytosolic proteins and membrane proteins. A score >-0.4 indicates probability for membrane association (the higher the score is, the greater the probability is).

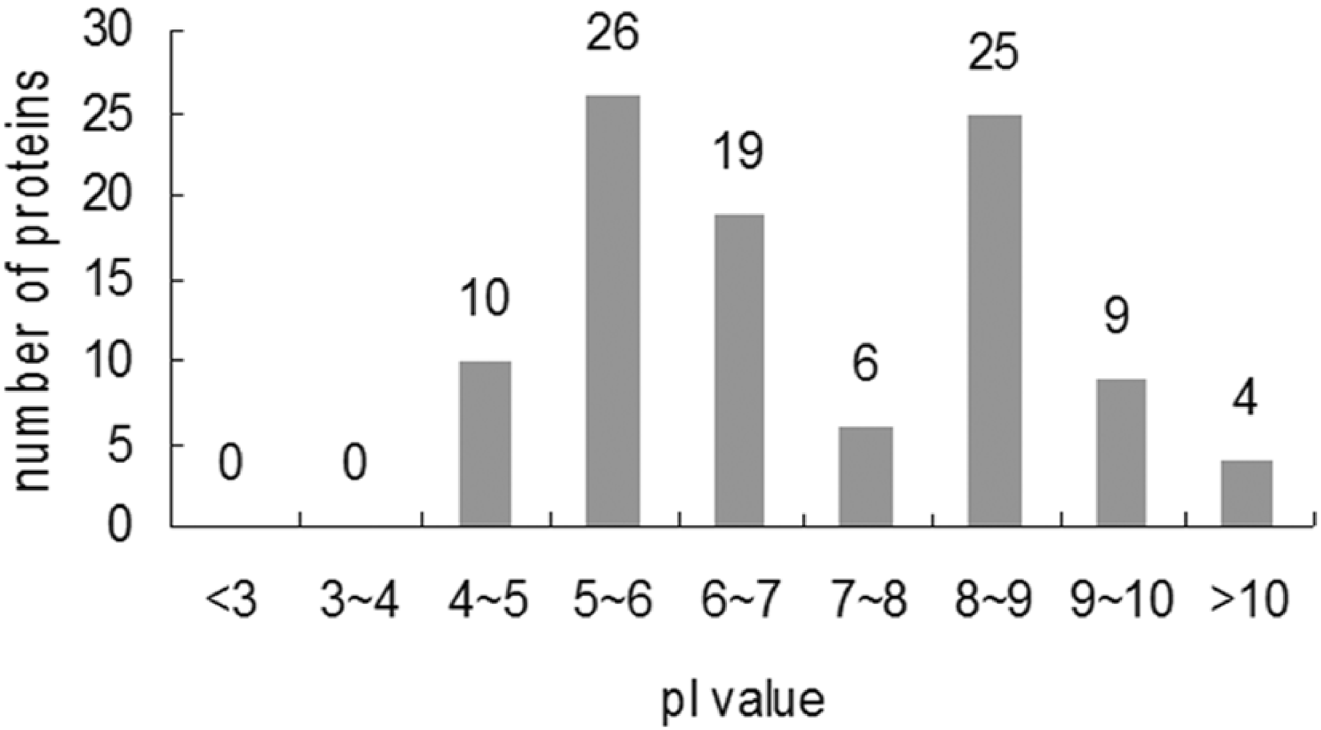

Supplemental Figure 4. Distribution of 1DE-derived proteins over the pI values.

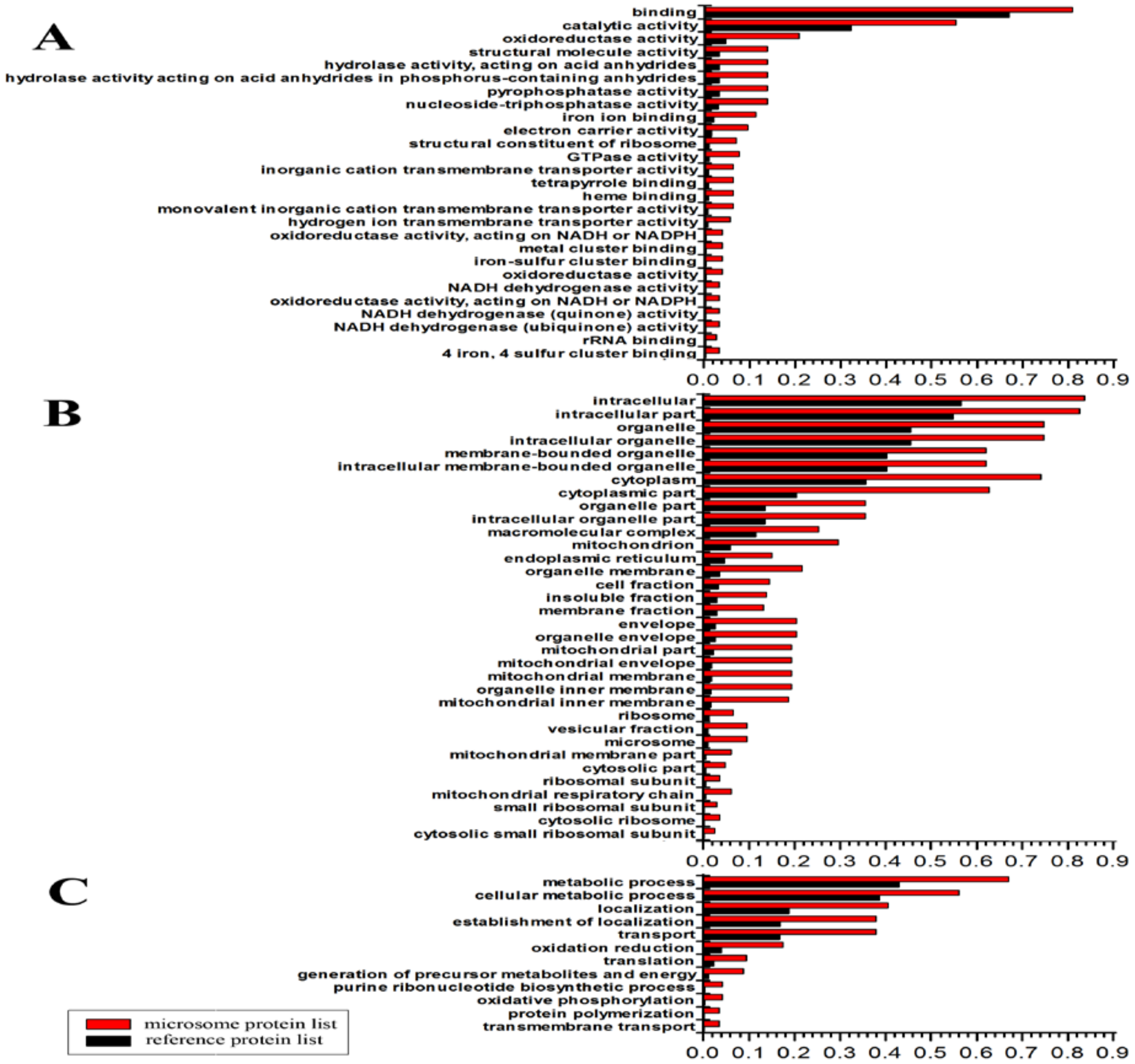

Supplemental Figure 5. Significant enrichment of GO terms for mouse liver microsome proteins (n = 259) that were derived from 1DE and 2DE strategies. The set of identified microsome proteins was compared with the reference list of IPI entries (provided by BinGO), and significantly over-represented GO terms ($P < 0.01$) are shown. The ratio shown is the number of microsome and reference IPI proteins annotated to each GO term divided by the number of microsome and reference IPI proteins linked to at least one annotation term within the indicated GO cellular component(A), molecular function(B), and biological process categories(C).
